# The transforming growth factor beta ligand TIG-2 modulates the function of neuromuscular junction and muscle energy metabolism in *Caenorhabditis elegans*

**DOI:** 10.3389/fnmol.2022.962974

**Published:** 2022-10-28

**Authors:** Xinran Cheng, Zhenzhen Yan, Zexiong Su, Jie Liu

**Affiliations:** ^1^Neuroscience Program, Department of Anatomy and Developmental Biology, Monash Biomedicine Discovery Institute, Monash University, Melbourne, VIC, Australia; ^2^Dr. Neher’s Biophysics Laboratory for Innovative Drug Discovery, State Key Laboratory of Quality Research in Chinese Medicine, Macau University of Science and Technology, Macao, Macao SAR, China

**Keywords:** *Caenorhabditis elegans*, TIG-2, locomotion, neuromuscular junction, acetylcholine, mitochondria

## Abstract

Deciphering the physiological function of TGF-β (the transforming growth factor beta) family ligands is import for understanding the role of TGF-β in animals’ development and aging. Here, we investigate the function of TIG-2, one of the ligands in *Caenorhabditis elegans* TGF-β family, in animals’ behavioral modulation. Our results show that a loss-of-function mutation in *tig-2 gene* result in slower locomotion speed in the early adulthood and an increased density of cholinergic synapses, but a decreased neurotransmitter release at neuromuscular junctions (NMJs). Further tissue-specific rescue results reveal that neuronal and intestinal TIG-2 are essential for the formation of cholinergic synapses at NMJs. Interestingly, *tig-2*(*ok3416*) mutant is characterized with reduced muscle mitochondria content and adenosine triphosphate (ATP) production, although the function of muscle acetylcholine receptors and the morphology muscle fibers in the mutant are comparable to that in *wild-type* animals. Our result suggests that TIG-2 from different neuron and intestine regulates worm locomotion by modulating synaptogenesis and neurotransmission at NMJs, as well as energy metabolism in postsynaptic muscle cells.

## Introduction

The TGF-β signaling pathway in mammals plays critical roles in cell differentiation, embryonic development, homeostasis and the pathogenesis of multiple diseases (Mulas et al., 2017; Liu et al., 2018). Given the evolutionarily conserved TGF-β signaling pathway from invertebrates to vertebrates, previous studies with different model organisms greatly promote better understanding of the physiological function of TGF-β signaling pathway in mammals. For example, research in *Drosophila* demonstrated that members in the TGF-β ortholog pathway are involved in synapse growth and stability, as well as homeostatic plasticity (Berke et al., 2013; Piccioli and Littleton, 2014).

*Caenorhabditis elegans* has been widely employed as a model organism to investigate the function of different signal transduction pathway, including TGF-β signaling, in the body size regulation and animals’ behavioral modulation (Gumienny and Savage-Dunn, 2018). According to homologous sequence analysis, five TGF-β ligands (*daf-7*, *dbl-1*, *unc-129*, *tig-2*, and *tig-3*), two type I receptors (*daf-1* and *sma-6*), and one type II receptors (*daf-4*) were identified in the *C. elegans* (Savage-Dunn and Padgett, 2017). Previous research showed that some ligands of TGF-β signaling are involved in locomotion regulation of *C. elegans* by regulating gene expression (Hilbert and Kim, 2017). For example, DAF-7 exhibits a male-specific expression pattern in the ASJ sensory neurons and modulates male exploration behavior in *C. elegans* (Hilbert and Kim, 2017).

TIG-2, one of the ligands in the TGF-β signaling pathway, has been shown to be involved in multiple physiological functions in *C. elegans*, such as neuronal guidance (Baltaci et al., 2022). Bone morphogenetics protein 8 (BMP8), the ortholog gene of *tig-2* in human, mainly expresses in developing skeletal and brain tissue (Simpson, 1756; Gumienny and Savage-Dunn, 2018), and is involved in the regulation of gene expression, embryogenesis, and tumorigenesis, while research with *Drosophila* demonstrated that glass bottom boat (*Gbb*), the ortholog gene of *tig-2* in flies, regulates synapse development by working as a retrograde signaling from muscles to neurons at NMJs (Berke et al., 2013). However, the function of TIG-2 at NMJs in *C. elegans* has never been studied.

In this study, we demonstrated the function of TIG-2 in the synapse formation and neurotransmission at NMJs, as well as muscle energy metabolism in *C. elegans*. Our results showed that TIG-2 modulates the movement of *C. elegans* in early adulthood through two different mechanisms. First, neuron- and intestine-derived TIG-2 is required to maintain the function and structure of cholinergic NMJs. Second, TIG-2 shapes the mitochondria content and the mitochondrial calcium concentration in body wall muscle cells, which in turn maintain the energy metabolism in muscle cells. Collectively, the study revealed that TIG-2 regulates *C. elegans* locomotion by modulating the morphological and neurotransmitter release at NMJs, as well as the energy generation in muscle cells in early adulthood.

## Materials and methods

### Worm maintenance

Nematodes were cultivated following previous standard procedures (Stiernagle, 2006). Briefly, worms were grown at 20°C on nematode growth media (NGM) plates. *Escherichia coli* (*E. coli*) OP50 was used as the food source, which was cultured overnight at 37°C in lysogenic buffer (LB) media prior to be seeded on NGM plates. All worms were synchronized by picking L4 stage animals and analyzed at the required age (Day 3). The wild-type reference strain was Bristol N2. *C. elegans* mutant strains (Table 1) used in this study were backcrossed with wild-type worms for at least three times. Tissue specific rescue lines were generated by the microinjection plasmids mix (Table 2) into the gonad of young adult *tig-2*(*ok3416*) mutant as previously described (Mello et al., 1991). Worms after injection were cultured until they produced offspring that can stabilize with fluorescence.

**TABLE 1:** Worm strains used in this study.

External strains		
Code	Strain	Source
MLJ59	*tig-2(ok3416)*	CGC
MLJ313	*tauIs44[Punc-129::mcherry::RAB-3]*	Hu Lab
MLJ229	*oxIs364[unc-17p::channelrhodopsin::mCherry + lin-15(+) + Litmus]*	CGC
MLJ268	*ufIs8[myo-3p::ACR-16::GFP]*	Francis Lab
MLJ244	*zcIs14[myo-3p::GFP(mit)]*	CGC
MLJ1160	*RW1596[myo-3(st386) V; stEx30(myo-3p::GFP::myo-3 + rol-6(su1006))]*	Neumann Lab
**Strains generated in Liu’s Lab**		
MLJ1127	*tig-2(ok3416);tauIs44[Punc-129::mcherry::RAB-3]*	
MLJ364	*tig-2(ok3416);ufIs8[myo-3p::ACR-16::GFP]*	
MLJ1101	*tig-2(ok3416);MLJEx045[tig-2fosmid;coel-1::GFP]*	
MLJ1102	*tig-2(ok3416); MLJEx046[Pmyo-3::tig-2::gfp]*	
MLJ1103	*tig-2(ok3416); MLJEx047[Pges-1::tig-2::gfp]*	
MLJ1104	*tig-2(ok3416); MLJEx048[Prgef-1::tig-2::gfp]*	
MLJ1130	*tig-2(ok3416);MLJEx045[tig-2fosmid;coel-1::GFP];tauIs44*	
MLJ1131	*tig-2(ok3416); MLJEx046[Pmyo-3::tig-2::gfp];tauIs44*	
MLJ1132	*tig-2(ok3416); MLJEx047[Pges-1::tig-2::gfp];tauIs44*	
MLJ1133	*tig-2(ok3416); MLJEx048[Prgef-1::tig-2::gfp];tauIs44*	
MLJ218	*MljIs001[Pmyo-3::mito::GCaMP3.0]*	
MLJ407	*tig-2(ok3416); MljIs001[Pmyo-3:mito:GCaMP]*	
MLJ408	*tig-2(ok3416); zcIs14[Pmyo-3:GFP(mito)]*	
MLJ371	*mcu-1(ju1154); MljIs001[Pmyo-3:mito:GCaMP]*	
MLJ1161	*tig-2(ok3416);mcu-1(ju1154); MljIs001[Pmyo-3:mito:GCaMP]*	
MLJ1153	*tig-2(ok3416);MljIs001[Pmyo-3:mito:GCaMP];MLJEx52[fosmid;Pmyo-2::mcherry]*	
MLJ1152	*tig-2(ok3416);mcu-1(ju1154); MljIs001[Pmyo-3:mito:GCaMP];MLJEx51[fosmid;Pmyo-2::mcherry]*	
MLJ1162	*tig-2(ok3416); zcIs14[Pmyo-3:GFP(mito)];MLJEx53[fosmid;Pmyo-2::mcherry]*	
MLJ1151	*oxIs364[unc-17p::channelrhodopsin::mCherry + lin-15(+) + Litmus];MLJEx50[Ptig-2::GFP]*	

**TABLE 2:** Plasmids mix used for microinjection.

Purpose	Strain injected	Plasmid/DNA	Concentration (ng/μl)
*tig-2* expression pattern	*oxIs364[unc-17p::channelrhodopsin::mCherry + lin-15(+) + Litmus]*	*Ptig-2::GFP*	20
		Bacterial DNA	130
Rescue of *tig-2* expression	*tig-2(ok3416)*	*tig-2* fosmid	2
		*Pcoel-1::GFP*	20
		Bacterial DNA	130
	*tig-2(ok3416)*	*Pmyo-3::tig-2::GFP*	20
		Bacterial DNA	130
	*tig-2(ok3416)*	*Pges-1::tig-2::GFP*	20
		Bacterial DNA	130
	*tig-2(ok3416)*	*Prgef-1::tig-2::GFP*	20
		Bacterial DNA	130
	*tig-2(ok3416);Pmyo-3::mito::GCaMP*	*tig-2* fosmid	2
		*Pmyo-2::mcherry*	10
		Bacterial DNA	140
	*tig-2(ok3416);mcu-1(ju1154);Pmyo-3::mito::GCaMP*	*tig-2* fosmid	2
		*Pmyo-2::mcherry*	10
		Bacterial DNA	140

### Quantification of locomotion

Worms used in the behavior test were grown under the standard condition (Sulston and Brenner, 1974). An automated worm-tracking system, WormLab (MBF bioscience, Williston, ND, USA), was used for tracking and behavioral analysis. Locomotion behavior assay was performed as described previously with minor modification (Liu et al., 2013). Briefly, NGM plates were freshly seeded with 20 μl fresh OP50 bacteria 10 mins prior to tracking, then six worms of each strain were randomly picked up from the cultured plates and transferred to the fresh seeded NGM plates. Worm images were recorded for 6 mins and the initial 1 min of tracking were not included for analysis. For each group, at least 30 worms were analyzed.

### Aldicarb-sensitivity assay

Aldicarb-sensitivity assay was performed as previously described with some modification (Mahoney et al., 2006). Aldicarb (33386, Sigma-Aldrich, Burlington, VT, USA) was dissolved into 70% ethanol to a concentration of 100 mM as a stock solution. NGM agar plates with aldicarb were prepared at least 1 day before the experiment. The aldicarb stock solution was diluted in the liquid NGM media to the final concentration of 0.5 mM. 4 ml of NGM agar was poured into a 60 mm petri dish and the plate was cooled at room temperature overnight. More than 60 L4 larval stage worms of each strain were prepared 3 days before the assay. Aldicarb plates were spread with a thin layer of OP50 bacteria 30 min prior to the assay, and 20 worms of each strain were transferred to aldicarb and control plates. Worm behaviors were checked every hour until all worms were completely paralyzed. The experiments were repeated at least three times under the same condition. Log-rank (Mantel-Cox) test was conducted to compare the survival time of the animals on the aldicarb plates.

### Confocal image and analysis

Nikon C1 upright confocal system equipped with Nikon 100× plan fluorite oil immersion objective was used for confocal image. Fluorescence green fluorescent protein (GFP) and mCherry were excited by diode argon 488 nm and DPSS 561 nm lasers, respectively. Worms from culture plates were washed in the M9 buffer, then immobilized with polystyrene microspheres (00876-15, Polybead, Warrington, WA, USA) and mounted on agarose pads (2%). A coverslip was carefully placed on the pad for image shooting.

The images of synapses were captured for the anterior body of worms, between head and vulva. Maximum projection images generated from acquired Z-stacks were used for analysis. To quantify the synapse puncta, the open-source image software Fiji (ImageJ) was used to count the total number of puncta and measure the length of the nerve cord. By dividing the cluster number with the length of nerve code, an average synapse number in every 100 μm nerve code was determined. For myosin fiber imaging, the analysis was performed following the previous publication (Teoh et al., 2019).

Mitochondria morphology assay was conducted according to the methods described in previous study (Giacomotto et al., 2013). Approximately 16 body-wall muscle cells per worm were observed, in which the cells in head and tail were excluded. The cells images were divided into three groups according to the different morphology of mitochondria (Giacomotto et al., 2013): (1) long interconnected mitochondrial networks were classified as tubular; (2) interconnected mitochondrial network along with some fragmented mitochondria were classified as intermediate; (3) short and fragmented mitochondria were classified as fragmented. The morphology categories analysis was performed in a double-blind manner.

For the muscle mitochondrial GFP imaging and muscle mitochondrial GCaMP imaging, the Nikon 10x plan achromat objective and the Nikon 40x plan fluorite objective was used, respectively. Linear adjustments of brightness and contrast were performed to the same degree for all imaging. To quantify the mitochondrial intensity, the head muscles of the worm were chosen, and the images were quantified with Fiji.

### Quantification of intensity of mitochondrial green fluorescent protein

The quantification of intensity of mitochondrial GFP was conducted according to the methods described in previous study (Katsyuba et al., 2018). The worms were prepared as follows: about 80 worms per group were washed off from the plates with M9 buffer. Before being transferred into a black-walled 96-well plate containing 50 μl of M9 buffer (20 worms per well), worms were washed with M9 buffer at least three times to remove residual bacteria. GFP fluorescence was measured using a CLARIOstar Plus Microplate reader (BMG Labtech) at excitation 485 nm and emission 520 nm. Each experiment was repeated at least three times.

### Adenosine triphosphate quantification

The procedure of ATP measurement was modified from the previous report (Mao et al., 2019). Day 3 worms were washed with M9 buffer three times before collecting. For each genotype strain, 80 worms were collected in 80 μl M9 buffer and quickly frozen in the liquid nitrogen. Worms were lysed for 15 mins at 90°C. Lysis solution was then centrifuged for 13,000 × *g* at 4°C for 10 mins, and supernatant was transferred into a new tube for subsequent assay. The ATP level was determined by ATP determination kit (A22066, Thermo Fisher Scientific, Waltham, MA, USA) according to the manufacturer’s instruction. The protein concentration was measured and normalized using Pierce™ BCA Protein Assay Kit (catalog number: 23227, Thermo Fisher Scientific, Waltham, MA, USA). CLARIOstar Plus Microplate reader (BMG Labtech) was employed for the ATP and BCA assay. At least three times repeat were conducted for each experiment.

### Electrophysiology

Electrophysiological recordings were performed on the dissected nematodes as previously described (Hu et al., 2012). An Olympus microscope (BX51WI) equipped with EPC-10 amplifier and Patchmaster software (HEKA) was used for whole-cell patch clamp recording. The current data was filtered at 2 kHz and sampled at 20 kHz. Both series resistance and membrane capacitance were compensated. The Sutter puller (P-1000, Sutter Instruments) was used to pull the borosilicate glass capillary (BF150-86-10, Sutter Instruments) to get a recording pipette with a resistance of 3–5 MΩ. The pipette solution contains 135 mM CH_3_O_3_SCs, 5 mM CsCl, 5 mM MgCl_2_, 5 mM EGTA, 0.25 mM CaCl_2_, 10 mM HEPES, and 5 mM Na_2_ATP (325 mOsm), and pH was adjusted to 7.2 with CsOH. The bath solution contains 140 mM NaCl, 5 mM KCl, 5 mM MgCl_2_, 1 mM CaCl_2_, 11 mM glucose, and 10 mM HEPES (330 mOsm), and pH was adjusted to 7.3 with NaOH. Dermabond glue (2-octyl cyanoacrylate, Ethicon) was used to attach worms to sylgard-coated coverslips. A small piece of cuticle of the worm body was then carefully peeled and glued to the coverslip to expose the muscle cells for recording. All experiments were performed at room temperature, and the body wall muscle cells were clamped at –60 mV. The frequency and amplitude of mPSCs were analyzed using Igor Pro (WaveMertrics, portland, OR, USA). A detection threshold in the initial automatic analysis was set at 5 pA.

### Statistical analysis

Statistical analysis was conducted by GraphPad Prism software (Dotmatics). The specific quantification and statistical parameters (error bars, *n* numbers, and *p*-values) were declared in each figure legend. Statistically significant was recognized when *p*-value was lower than 0.05.

## Results

### *tig-2* mutant exhibits reduced locomotion speed

**FIGURE 1:**
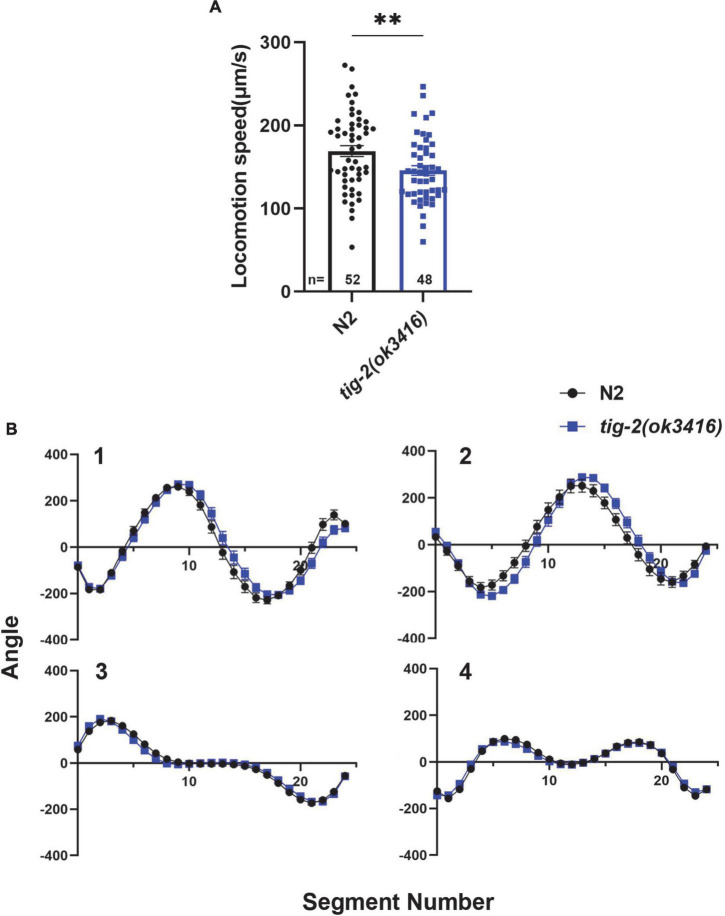
*tig-2* mutant displays lower locomotion speed than *wild-type* worms. **(A)** *tig-2*(*ok3416*) mutant has a lower locomotion speed is lower in on day 3 compared to wild-type animals (*p* = 0.0099). **(B)** The four eigenworms derived from *wild-type* worms and *tig-2*(*ok3416*) mutants are similar. A student’s *t*-test was performed to determine the significance of differences in locomotion speed. Error bars represent SEM. ^**^p < 0.01.

To investigate the function of TIG-2 ligand at NMJs in *C. elegans*. Here, we first checked whether TIG-2 modulate the locomotion of *C. elegans*. By monitoring the locomotion of day 3 worms, we found that *tig-2*(*ok3416*) mutant showed lower locomotion speed than wild-type worms (Figure 1A). By comparing the four eigenworms, which provide essentially complete description of worms’ posture (Brown et al., 2013), between *tig-2* mutant and *wild-type* worms, we found that *tig-2* mutant and *wild-type* animals exhibited similar locomotion patterns (Figure 1B). These findings indicated that *tig-2* loss-of-function mutation induces a decreased locomotion speed in worms but had no effect on the body postures while worms were moving.

### *tig-2* mutant has decreased acetylcholine release but increased density of cholinergic synapse at neuromuscular junctions

Since *C. elegans* locomotion is directly regulated by the function of NMJs, we next compared the structure and *in vivo* neurotransmission at NMJs between *tig-2*(*ok3416*) mutant and wild-type animals. Since *tig-2* was not expressed in GABAergic motor neurons (Supplementary Figure 1), only the morphology and the function of excitatory cholinergic NMJs were investigated.

**FIGURE 2:**
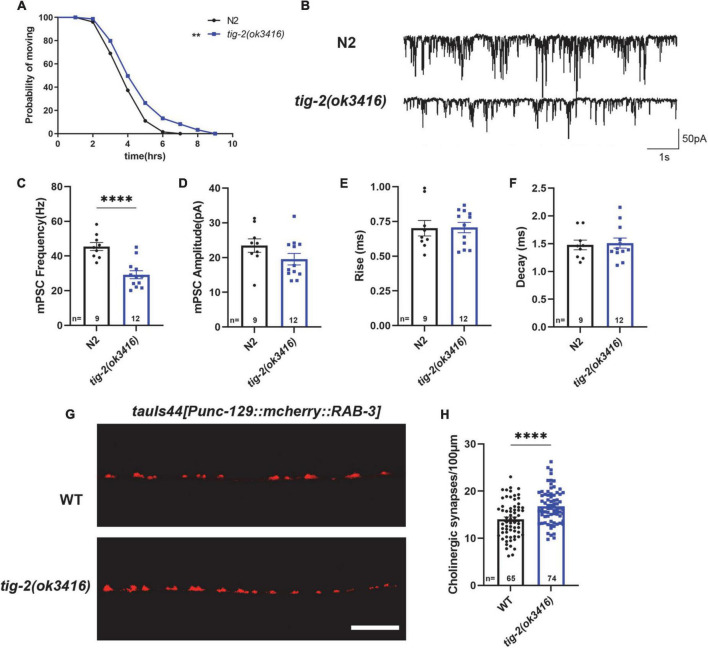
*tig-2* mutant has decreased acetylcholine (ACh) release but increased density of cholinergic synapses. **(A)** Comparison of aldicarb induced paralysis of *tig-2*(*ok3416*) mutant and wild-type worms. In early adulthood, *tig-2*(*ok3416*) mutant is more aldicarb-resistant than wild-type worms (*p* = 0.0055), indicating that *tig-2* mutant releases less ACh on day 3. *n* > 60. **(B)** Representative traces of miniature post-synaptic currents (mPSCs) on day 3. **(C,D)** The frequency of mPSC in *tig-2*(*ok3416*) mutant is lower than that in wild-type worms (*p* < 0.0001), but the amplitude of mPSC is similar between *tig-2* mutant and wild-type worms (*p* = 0.1384). **(E,F)** Quantifications of the 25–75% rise time and decay time of mPSCs. The data shows that no significant difference between wild-type and *tig-2*(*ok3416*) mutant. **(G)** Images of the cholinergic synapses on the nerve code. **(H)** Images of *tauIs44* strain show that the density of cholinergic synapses on the nerve cord is increased in *tig-2*(*ok3416*) mutants (*p* < 0.0001). A student’s *t*-test was performed to determine the significance of differences in mean density. Error bars represent SEM. ^**^p < 0.01, ^****^p < 0.0001.

We first checked the acetylcholine (ACh) release at *C. elegans* NMJ by performing aldicarb assay on both *tig-2*(*ok3416*) mutant and wild-type worms. Aldicarb is an inhibitor of acetylcholinesterase (AChE), which inhibits the hydrolysis of ACh. Aldicarb treatment induced ACh accumulation in the synaptic cleft results in the excessive activation of ACh receptors (AChRs) in body wall muscle cells, which in turn generates continuous muscle contraction, body paralysis, and ultimately death of *C. elegans* (Mahoney et al., 2006). In this study, we found that the mean time (4.8 h) for aldicarb-induced paralysis in *tig-2*(*ok3416*) mutant was significantly longer than that (4.1 h) in wild-type animals. The maximum time for all worms to become paralyzed was also significantly extended in the *tig-2* mutant (Figure 2A). This experiment supported that the accumulation of ACh at NMJs in *tig-2*(*ok3416*) is slower than that in *wild-type* worms, suggesting a reduced ACh release at NMJs in *tig-2*(*ok3416*) mutant.

To further confirm the function of TIG-2 on neurotransmission at NMJs, neurotransmission was analyzed by recording the transient miniature post-synaptic currents (mPSCs) at NMJs (Figure 2B). The frequency of spontaneous mPSCs indicates the number of vesicles releasing at the synapse, and the amplitude of mPSCs depends on the number of neurotransmitters within a single vesicle and the function of postsynaptic receptors (Isaac et al., 1995; Liao et al., 1995). Compared with wild-type worms, *tig-2*(*ok3416*) mutant displayed a reduced frequency of mPSCs on day 3 (Figure 2C), consistent with the decreased ACh release and the lower locomotion speed found in the mutant. However, the amplitude, as well as the 25–75% rise time and the decay time of mPSCs in *tig-2*(*ok3416*) mutant and wild-type worms were similar (Figures 2D–F), indicating that *tig-2* loss of function does not induce changes in the number of neurotransmitters in each vesicle, and the sensitivity of postsynaptic receptors of the mutant was similar to that of wild-type worms.

Decreased neurotransmitter release may result from a reduction in the number of presynaptic axons or a diminished ability of individual synapses in synaptic vesicle neurotransmitter release. Therefore, we further asked whether the number of synapses on the nerve cord of *tig-2* mutants changes by visualizing cholinergic NMJs with presynaptic vesicle marker RAB-3 (*tauIs44*[*Punc-129:mcherry:RAB-3*]). Surprisingly, the mutant worm exhibited an increase in cholinergic synapse puncta (Figures 2G,H). Decreased ACh release and increased cholinergic synapse in *tig-2*(*ok3416*) could be a compensatory effect in *C. elegans* NMJs, through which animals can generate more synapses to offset any deficiency in ACh release, which also indicates the import function of TIG-2 in synaptic homeostasis.

### *tig-2* mutant and *wild-type* worms have similar density and function of postsynaptic receptors

**FIGURE 3:**
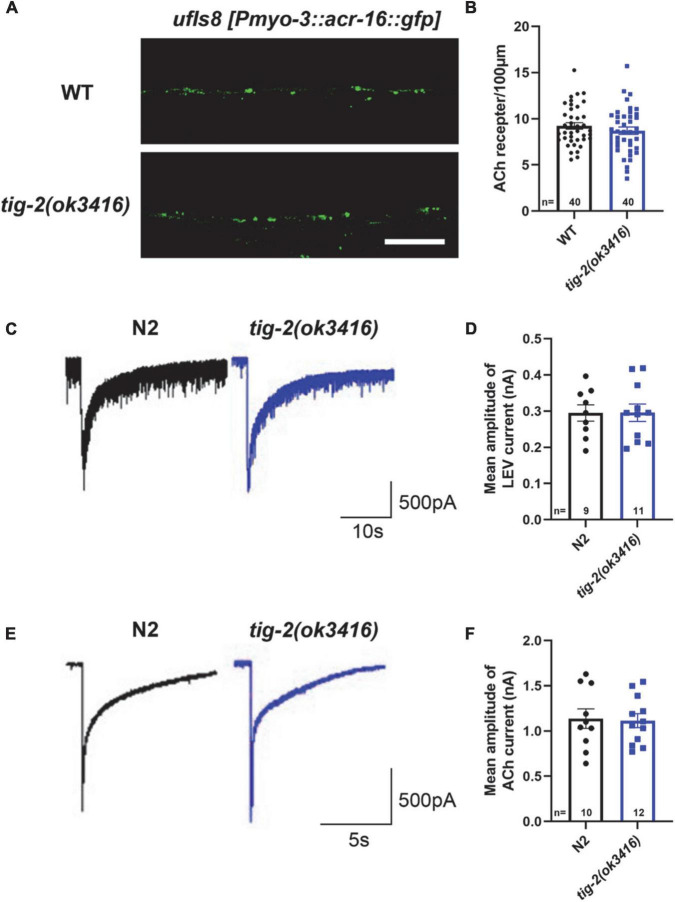
*tig-2* mutants and *wild-type* worms display similar density and function of postsynaptic AChRs. **(A)** Images of the post-synapse on the dorsal nerve cord. Scale bar is 10 μm. **(B)** Quantification of the density of post-synapses. The density of postsynaptic AChRs is unchanged in *tig-2*(*ok3416*) mutant (*p* = 0.3327). **(C,E)** Postsynaptic currents at NMJs evoked by levamisole **(C)** or ACh **(E)**. **(D,F)** Levamisole-evoked current (*p* = 0.9811) and ACh-evoked current (*p* = 0.8671) are similar in *tig-2*(*ok3416*) mutants and *wild-type* worms. A Student’s *t*-test was performed for each statistical analysis. Error bars represent SEM.

We next asked whether the number and function of postsynaptic receptors in muscle cells of *tig-2* mutants differed from those in *wild-type* worms. To determine the density of muscle receptors, *tig-2*(*ok3416*) mutant were crossed with *ufIs8*[*myo-3p:ACR-16:GFP*], in which acetylcholine receptor ACR-16 is marked with GFP. Calculating the number of fluorescent puncta showed that *tig-2*(*ok3416*) mutant has comparable fluorescent density of acr-16:GFP as wild-type worms (Figures 3A,B).

Although the density of cholinergic receptors was unaltered in *tig-2* mutant, it was unclear whether the function of postsynaptic receptors was impaired. Previous research revealed that disruption of either L-AChRs or N-AChRs results in negligible locomotory defects in *C. elegans*, but disruption of both leads to complete paralysis (Francis et al., 2005; Touroutine et al., 2005). To check whether the functional changes of postsynaptic cholinergic receptors present in *tig-2* mutant, the drug-induced inward currents were recorded by directly perfusing AChRs agonists, 100 μM levamisole and 1 mM ACh, to muscles. No obvious changes in drug-induced currents were found in *tig-2*(*ok3416*) mutant (Figures 3C–F). This result suggests that the expression level of functional AChRs in *tig-2*(*ok3416*) mutants is comparable to that in wild-type worms (Figure 3D).

### Neuronal and intestinal TIG-2 play important roles in locomotion and synapse formation

**FIGURE 4:**
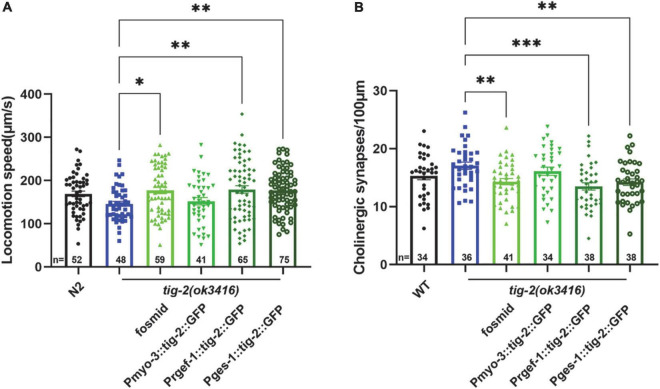
Neuronal or intestinal expression of *tig-2* abolishes locomotion speed defects and decreased density of cholinergic synapse in *tig-2*(*ok3416*). **(A)** Locomotion speed on day 3 in *tig-2*(*ok3416*) mutants and tissue-specific *tig-2* rescue alleles. Green columns indicate transgenic lines that carry plasmids restoring the expression of *tig-2* in muscle cells (*p* = 0.9768), neurons (*p* = 0.0060), intestine (*p* = 0.0089) and globally rescue (*p* = 0.0139). Neuronal TIG-2 and intestinal TIG-2 abolish the decrease in locomotion speed in the mutant. **(B)** Qualification of the density of cholinergic synapse in *tig-2*(*ok3416*) mutants and tissue-specific *tig-2* rescue alleles. Neuronal tig-2 (*p* = 0.0003) and intestinal tig-2 (*p* = 0.0062) suppress the increase of presynaptic puncta. *p*-values were calculated by one-way ANOVA with multiple comparisons. Error bars represent SEM. *p < 0.05, ^**^p < 0.01, ^***^p < 0.001.

Because *tig-2* is expressed widely in neurons, muscles, and the intestine of *C. elegans*, we therefore further investigate the function of tissue-specified expression of TIG-2 in animals’ locomotion. Driven by tissue specific promoter, tig-2 cDNA was expressed in either the global worm body, body wall muscles, neurons, or intestine. The locomotion speed recorded on day 3 revealed that the globally restoring expression of *tig-2* cDNA rescued the decreased movement in *tig-2*(*ok3416*) mutants. Interestingly, specifical restoring the expression of *tig-2* in either neurons or intestine, but not muscle, increased locomotion speed to the same level as in *wild-type* worms, suggesting that TIG-2 in neurons and intestine, but not in muscle cells, regulates the locomotion speed of *C. elegans* (Figure 4A).

To further understand the tissue-specific function of TIG-2 in synapse formation, *tig-2* cDNA driven by tissue specific promoter was injected into *tig-2*(*ok3416*)*;tauIs44*[*Punc-129:mcherry:RAB-3*]. In accordance with the results for locomotion speed, specifically restoring the expression of *tig-2* in neurons and intestine eliminated the changes in the number of synapses in *tig-2* mutant. To be more specific, when the expression of *tig-2* was restored in neurons and intestine in the *tig-2*(*ok3416*) mutants, the number of cholinergic synapses decreased significantly, whereas expressing *tig-2* specifically in muscle cells in the mutant had no impact on the formation of cholinergic synapses (Figure 4B). Taken together, these results suggest that neuronal and intestinal expression of *tig-2* is essential for regulating locomotion speed and synaptic formation in *C. elegans*.

### *tig-2* mutant shows intact morphology of body wall muscles

**FIGURE 5:**
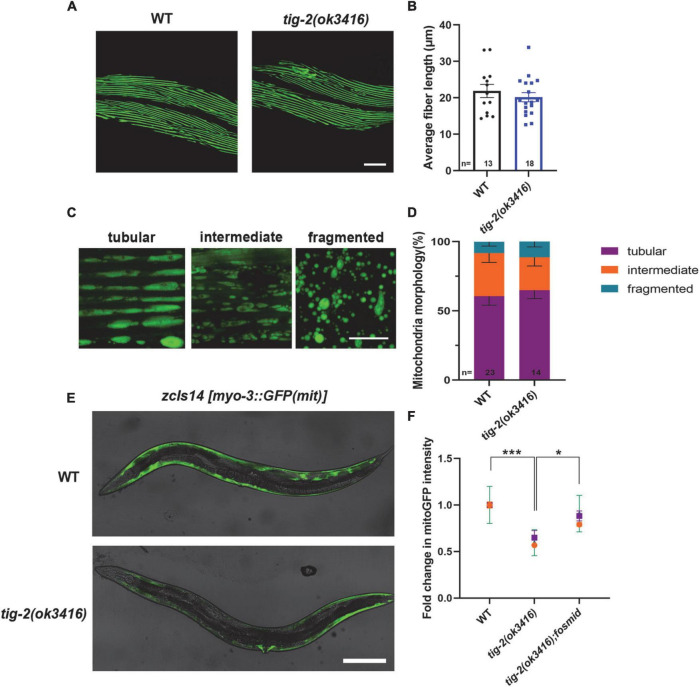
*tig-2* mutant has decreased mitochondrial content and mitochondrial calcium. **(A)** Images represent myosin fibers in body wall muscles. Scale bar is 20 μm. **(B)** Quantification of average muscle fiber length of individual worms. *tig-2*(*ok3416*) mutants and *wild-type* worms exhibit similar muscle fiber morphology (*p* = 0.4244). A student’s *t*-test was performed. **(C)** Representative images of mitochondria in body wall muscles showing three classes of cells. Scale bar is 5 μm. **(D)** Quantification of three classes of muscle cells in *wild-type* worms and *tig-2*(*ok3416*) mutants. Data from day 3 adult worms suggested similar mitochondrial morphology in *tig-2* mutants and *wild-type* worms. *p*-values were calculated by two-way ANOVA. **(E)** Representative images of mitochondria targeted green fluorescent protein (GFP) expressed in body wall muscles. Scale bar is 200 μm. **(F)** Quantification of the intensity of mitoGFP in muscle cells. The intensity in *tig-2*(*ok3416*) is lower than in *wild-type* worms (*p* = 0.0007), indicating fewer mitochondria content in *tig-2* mutants. All data are normalized to *wild-type* worms. *n* = 3 technical replicates. *p*-values were calculated by two-way ANOVA. Error bars represent SEM. *p < 0.05, ***p < 0.001.

*Caenorhabditis elegans* locomotion is accomplished by rhythmic contraction and relaxation of body wall muscles (Han et al., 2015). Although *tig-2*(*ok3416*) has normal expression and function of postsynaptic receptors and observed locomotion defect in *tig-2*(*ok3416*) could result from the functional defect in body wall muscle cells. We therefore crossed *tig-2*(*ok3416*) mutant with the strain expressing myo-3:GFP in muscle cells (*RW1596*[*myo-3*(*st386*) *V;stEx30*(*myo-3p:GFP:myo-3* + *rol-6*(*su1006*))]) (Figure 5A) to checked the morphology of muscles myosin following Jean et al.’s protocol (Teoh et al., 2019). The average length of muscle cell fiber branches in wild-type nematodes and *tig-2*(*ok3416*) mutants was found to be similar (Figure 5B), indicating that TIG-2 has no effect on the myosin morphology in body wall muscle cells.

### *tig-2* mutant has decreased mitochondrial content

Muscle contraction is an energy consuming process, which depends on the function of mitochondria in muscle cells. We next investigated whether energy production in muscle cells is reduced in *tig-2*(*ok3416*) mutant. We checked the mitochondrial morphology by crossing *tig-2*(*ok3416*) mutant with a strain *zcIs14*[*myo-3p:GFP*(*mit*)] expressing a mitochondria-targeted GFP under the body wall muscle *myo-3* promoter. The mitochondria in body wall muscle cells were classified into three types – tubular, intermediate, and fragmented (Figure 5C). Our result showed there was no significant difference between *tig-2*(*ok3416*) mutant and *wild-type* worms in mitochondrial morphology (Figure 5D), because the percentage of each class was similar between mutant and *wild-type* worms. However, the results showed that the mitochondria-targeted fluorescence intensity of muscle cells in *tig-2*(*ok3416*) mutants was nearly 50% lower than that of muscle cells in *wild-type* worms, indicating that the mitochondrial content of muscle cells was lower in *tig-2* mutants (Figure 5E). The reduction was rescued by expressing *tig-2* fosmid in *tig-2*(*ok3416*) mutant (Figure 5F), which further supported the function of TIG-2 in muscle mitochondrial content regulation.

### TIG-2 modulates adenosine triphosphate generation through MCU-1 mediated muscle mitochondrial calcium homeostasis

**FIGURE 6:**
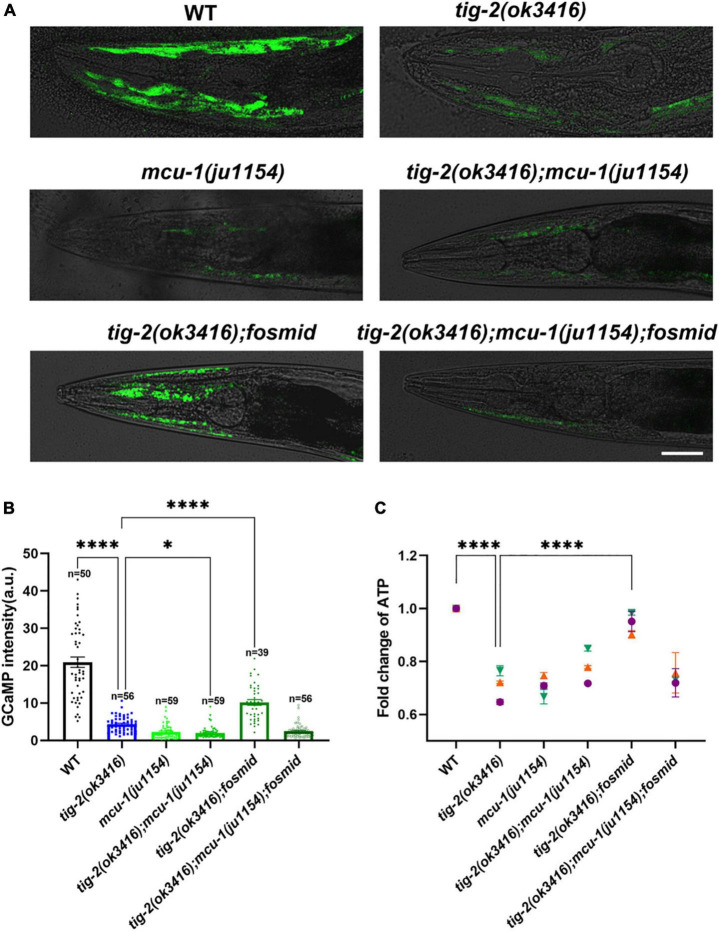
The content of mitochondrial Ca^2+^ and adenosine triphosphate (ATP) production are lower in *tig-2* mutants than in *wild-type* worms. **(A)** Representative images of GCaMP3 expression. Scale bar = 10 μm. **(B)** Quantification of mitochondrial Ca^2+^. The mitochondrial calcium content in *tig-2*(*ok3416*) mutant, *mcu-1*(*ju1154*) mutant and *tig-2; mcu-1* double mutants is similar, and much lower than that in wild-type worms (*p* < 0.0001). Restoring the expression of *tig-2* eliminates the decreased Ca^2+^ level in *tig-2* signal mutants (*p* < 0.0001), but not in *tig-2 mcu-1* double mutants (*p* = 0.1268). *p*-values were calculated by one-way ANOVA with multiple comparisons. **(C)** Relative ATP levels in worms expressing mito:GCaMP3 in their muscle cells. Data represents results from three independent tests. Each test was done in triplicate wells. *n* = 3 technical replicates. *p*-values were calculated by two-way ANOVA with multiple comparisons. Error bars represent SEM. ***p < 0.001, ****p < 0.0001.

Decreased locomotion speed and mitochondrial content in muscle cells of *tig-2*(*ok3416*) prompted us to investigate the energy production in the mutant. Normalizing the ATP level of *tig-2*(*ok3416*) mutant to that of wild type worms revealed that *tig-2* mutant generated less ATP, and this deficiency was eliminated after restoring the expression of *tig-2* in mutants (Figure 6C).

Previous research revealed that mitochondrial Ca^2+^ is essential for the regulation of mitochondrial function and ATP generation (García-Casas et al., 2021). To check whether the decrease in ATP results from the decline in mitochondrial Ca^2+^, the mitochondrial Ca^2+^ level in muscle cells was analyzed by monitoring the intensity of GCaMP, a genetic fluorescent mitochondrial calcium indicator. An integrated strain *MljIs001*[*Pmyo-3:mito:GCaMP3.0*], which specifically expresses the GCaMP indicator in body wall muscles mitochondria, was used for this assay. Since the fluorescence signal is most obvious in the head muscles, all images used for analysis were captured from the heads of worms (Figure 6A). Strikingly, the fluorescence intensity of GCaMP was 5-fold lower in *tig-2*(*ok3416*) mutants than that of *wild-type* worms (Figure 6B), supporting a significantly lower concentration of mitochondrial Ca^2+^ in *tig-2* mutant.

The mitochondrial Ca^2+^ level is balanced by mitochondrial Ca^2+^ influx and efflux, in which mitochondrial calcium uniporter (MCU) plays a key role (Baughman et al., 2011; De Stefani et al., 2011). Previous research with *C. elegans* demonstrated the fast mitochondrial Ca^2+^ uptake in wild-type worms is much larger than that in MCU-defective worms [*mcu-1*(*ju1154*)], which supported conservation of *mcu-1* function in mitochondrial Ca^2+^ uptake (Álvarez-Illera et al., 2020). To determine whether the reduction of mitochondrial Ca^2+^ induced in *tig-2* mutant depend on MCU, we crossed *tig-2*(*ok3416*) with *mcu-1*(*ju1154*) and rechecked the concentration of mitochondrial calcium. As expected, MCU-defective worms had low mitochondrial Ca^2+^ level relative to the control. The double-mutant worms displayed comparable level of mitochondrial Ca^2+^ as either *tig-2* or *mcu-1* single mutant, suggesting that the role of TIG-2 in mitochondrial Ca^2+^ modulation depends on the function of MCU (Figure 6B). This was further supported by that restoring the expression of *tig-2* increased the mitochondrial Ca^2+^ level in *tig-2*(*ok3416*) mutant but had no effect on the *tig-2*(*ok3416*)*; mcu-1*(*ju1154*) double mutant. These results indicate that *tig-2* mutant has a lower concentration of mitochondrial Ca^2+^ in muscle cells than *wild-type* worms, and the size of the reduction depends on the function of MCU-1 (Figure 6B).

The finding of the dependence on MCU-1 for regulation of mitochondrial Ca^2+^ content raised the question of whether the observed decreased ATP level in *tig-2*(*ok3416*) also depend on the function of MCU-1. ATP quantification showed that loss-of-function mutation of *mcu-1* produced less ATP than *wild-type* worms (Figure 6C). As expected, ATP content was also lower in *tig-2*(*ok3416*) mutant and *tig-2*; *mcu-1* double mutant than in *wild-type*, suggesting that the defect in ATP production induced by *tig-2* loss-of-function was dependent on the function of MCU-1. When the expression of *tig-2* was restored in the *tig-2*(*ok3416*)*; mcu-1*(*ju1154*) double mutants, the mean ATP content was only about 70% of that in *wild-type* worms, whereas in the *tig-2*(*ok3416*) mutants, the ATP level was fully rescued by restoring the expression of *tig-2*. These results, combined with mitochondrial Ca^2+^ quantification, further demonstrated that TIG-2 modulated ATP production in *C. elegans* depends on the function of MCU-1.

## Discussion

### The effect of TIG-2 on neuromuscular junctions

Neuronal-muscular synaptic connection is a fundamental physiological unit of the motor system, which also regulate the locomotion in nematode *C. elegans*. However, much knowledge of the underlying mechanisms of synaptic formation and function remains to be discovered. Our research demonstrated that TIG-2, one of the ligands of TGF-β signaling pathway, regulates the locomotion speed by affecting the excitatory synapse formation and synaptic ACh release at NMJs in *C. elegans*.

The TGF-β family has been shown to influence many aspects of neural development and synapse formation in different species. For example, the DAF-7/TGF-β signaling pathway in *C. elegans* regulates the synaptic abundance of GLR-1, the AMPA-type glutamate receptor, and mutations of DFA-7 affect spontaneous locomotion in *C. elegans* (McGehee et al., 2015), and *Gbb* in *Drosophila* promotes NMJ synapse formation, while BMP7 augments synapse formation in the cultured neurons of mammals (Withers et al., 2000; Aberle et al., 2002). In addition, some members of the TGF-β family promote synaptic transmission. Early research has claimed that TGF-β1 encourages long-term rises in neuronal excitability in *Aplysia* (Chin et al., 2006), and that TGF-β2 treatment of cultured hippocampal neurons decreases short-term synaptic depression (Fukushima et al., 2007). Another member of the TGF-β superfamily, activin, has been reported to improve excitatory synaptic transmission, and suppress inhibitory synaptic transmission in cultured rat hippocampal neurons (Kurisaki et al., 2008; Krieglstein et al., 2011). In this study, by combining behavioral analysis and morphological analysis with electrophysiological recording at NMJs, *tig-2* mutants were found to exhibit less ACh release but more cholinergic synapses than *wild-type* worms. Consequently, it was thought that these opposite changes in the neurotransmitter release and synaptic formation might be a compensatory effect. Because the *tig-2* mutant worms display lower locomotion speed than *wild-type* worms, it was speculated that the *tig-2* loss-of-function mutation reduced excitatory neurotransmission, so that cholinergic synapse undergoes compensatory growth to promote ACh release. A compensatory negative feedback mechanism called homeostatic synaptic plasticity is a key means for animals to maintain neuronal network stability (Turrigiano and Nelson, 2000; Turrigiano, 2008). In accordance with previous research, homeostatic synaptic plasticity cannot only reduce synaptic strength to prevent excessive excitation when excitability is increased, but increase synaptic strength in the case of chronic activity inhibition to prevent unnecessary synaptic silencing and loss (Vitureira and Goda, 2013), suggesting the function of TIG-2 in synaptic plasticity modulation.

Neurotransmitter release from presynaptic motor neuron is a typical synaptic vesicle exocytosis, which is broadly divided into neurotransmitter uploading to synaptic vesicle, vesicle docking/priming, and vesicle fusion (Südhof and Rizo, 2011). Multiple presynaptic proteins are involved in the control of vesicle formation and fusion events to ensure normal neurotransmitter release and neuronal communication (Rizo, 2018; Brunger et al., 2019). Although the *tig-2* loss-of-function mutation has been proved to inhibit ACh release in *C. elegans*, whether TIG-2 modulates vesicle formation, transfer and/or exocytosis remains to be understood.

Research revealed that the ortholog gene of *tig-2* in *Drosophila*, *Gbb*, promotes the development of presynaptic boutons, which acts as a retrograde signal from muscle cells (McCabe et al., 2003). The type II TGF-β receptor *wit* is involved in this signaling pathway (Aberle et al., 2002). Therefore, one unsolved problem is whether TIG-2 in *C. elegans* acts as a retrograde signal like *Gbb*. To address this problem, tissue-specific rescue experiments were conducted in *C. elegans* to determine the locomotion speed and to observe the presynaptic puncta on the nerve cord. This work showed that the locomotion changes are restored by expressing *tig-2* in either neurons or intestine in the mutant, but not muscle cells, indicating that muscular TIG-2 has no effect on locomotory behavior. In addition, expressing *tig-2* in neurons and intestine led to synapse numbers like those in *wild-type* worms, whereas expressing *tig-2* in muscle cells did not restore the changes in cholinergic synapses, conforming that the function of TIG-2 from neurons and intestine, rather than muscle cells, in animal’s behavioral modulation. Therefore, TIG-2 at NMJs in *C. elegans* acts as an anterograde signal, from neurons to muscles, not a retrograde signal, to regulate synaptic formation, which supports functional diversity of TGF-β signaling in different species.

### The role of TIG-2 in mitochondrial calcium regulation

Recent research supports that TGF-β modulates cellular metabolism by regulating gene expression and mitogenesis (Liu and Chen, 2022). For example, *Smad3* ablation, which blocks TGF-β signaling in mice, enhance mitochondrial biogenesis in adipocytes and promote fatty acid oxidation (Liu and Chen, 2022). However, the molecular mechanisms of the role TGF-β signaling in bioenergetically modulation, especially in mitochondrial ATP synthesis is not clear.

Our research demonstrated the function of TIG-2 in energy metabolism in *C. elegans*. Reduced mitochondrial density and mitochondrial calcium level were found in muscle cells of *tig-2* mutant, which may contribute to the observed lower ATP level in *tig-2*(*ok3416*). Interestingly, TGF-β is also involved in mitogenesis in cultured vascular smooth muscle cells and intracellular calcium homeostasis (Weiss et al., 1995). Our study also demonstrated that TIG-2 modulates mitochondrial calcium homeostasis *via* a MCU dependent pathway. However, the physiological basis for the function of TIG-2 in mitochondrial calcium modulation is not clear. Given the role of TGF-β in modulating gene expression, it is possible that the expression of MCU-1 changes in *tig-2*(*ok3416*). TGF-β has also been shown to regulate intracellular calcium homeostasis by modulating the physiologic function of calcium permeable channels. For example, TGF-β1 stimulate *p38* MAPK, which in turn enhances intracellular calcium concentration *via* the increased expression of Ca1.2 channel in mice cortical neurons (Li et al., 2022). The physiological function of MCU is also modulated by different mechanisms. For example, transcription factor cyclic adenosine monophosphate response element (CREB) modulates MCU abundance by binding to the MCU promoter (Shanmughapriya et al., 2015), while Ca^2+^/calmodulin kinase II (CaMKII) enhances MCU activity through the phosphorylation of the NTD domain of MCU (Joiner et al., 2012). Therefore, TIG-2 may shape the mitochondrial calcium by modulating the expression of specific intracellular molecules, which in turn modifies the function of MCU-1. Another possible explanation for the function of TIG-2 in mitochondrial calcium modulation is that TIG-2 may modulate the endoplasmic reticulum (ER)-mitochondrial interaction, which regulates the calcium transportation from ER to mitochondria (Pacher et al., 2008). For instance, TGF-β has been reported to uncouple the ER-mitochondrial calcium transfer in arteriolar smooth muscle cells (Pacher et al., 2008). It is worth to check whether the similar mechanism also contribute to the function of TGF-β in *C. elegans*.

## Data availability statement

The raw data supporting the conclusions of this article will be made available by the authors, without undue reservation.

## Author contributions

XC and JL designed the experiments and wrote the manuscript. XC carried out the behavioral analysis, aldicarb assay, confocal imaging, and ATP analysis. ZY performed the electrophysiology recording. ZS assisted in the double-blind assay. All authors contributed to the article and approved the submitted version.

## Supplementary material

Supplementary Figure 1 *tig-2* expressed in cholinergic motor neurons on the nerve cord. **(A)** Confocal images of transgenic worms that expressed *Ptig-2:GFP*. **(B)** Confocal images of transgenic worms that expressed *Punc-17:ChR:mcherry*. **(C)** Expression was colocalized in the cholinergic neurons. Scale bar=20 μm.
