# Supplementary figures and images for: The transforming growth factor beta ligand TIG-2 modulates the function of neuromuscular junction and muscle energy metabolism in Caenorhabditis elegans
Source: Front Mol Neurosci. 2022 Oct 28;15:962974. doi: 10.3389/fnmol.2022.962974 (PMC9650414; doi:10.3389/fnmol.2022.962974)

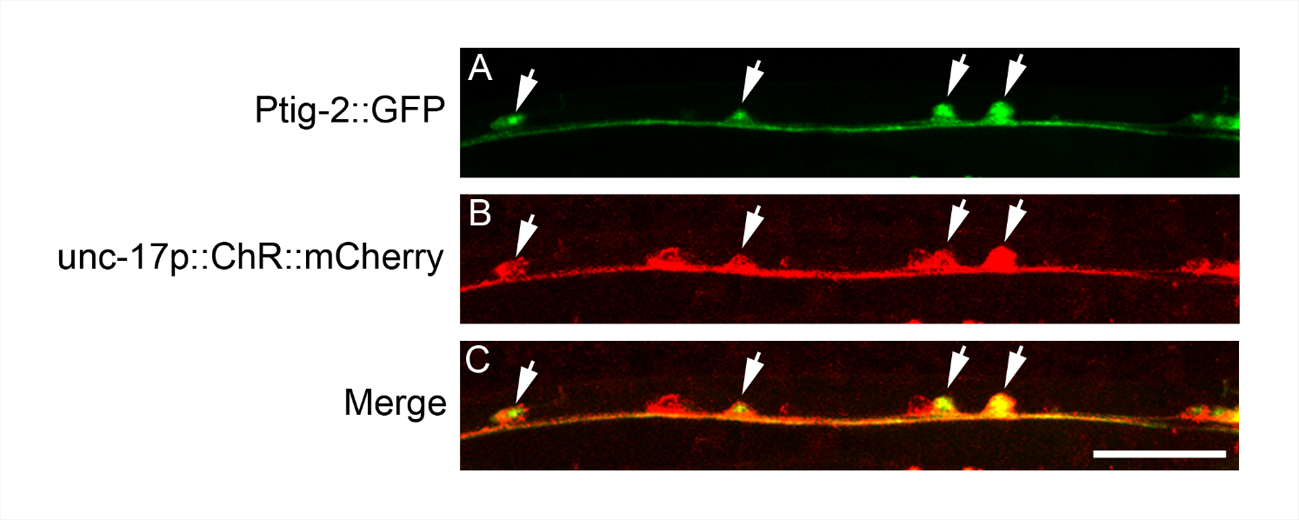

Supplement: Supplementary Figure 1 — tig-2 expressed in cholinergic motor neurons on the nerve cord. (A) Confocal images of transgenic worms that expressed Ptig-2:GFP. (B) Confocal images of transgenic worms that expressed Punc-17:ChR:mcherry. (C) Expression was colocalized in the cholinergic neurons. Scale bar=20 μm. [file Image_1.TIF]
